# Multicentric Disappearing Bone Disease treated with Arthroplasty

**DOI:** 10.5704/MOJ.1611.004

**Published:** 2016-11

**Authors:** CK Chan, Razif-MA Mohamed, AA Azlina, MM Azhar

**Affiliations:** NOCERAL, Department of Orthopaedic Surgery, Faculty of Medicine, University of Malaya, Petaling Jaya, Malaysia

**Keywords:** Disappearing bone disease, arthroplasty, Gorham Disease

## Abstract

Multicentric disappearing bone disease, or Gorham disease, is a rare entity. A middle age woman, presented to us with left sided antalgic gait and severe bony deformity of her left knee. Radiograph revealed massive bone defect of the medial condyle of the left tibia with subluxation of the knee joint. She was scheduled for knee replacement in six months. However, she developed another lesion over the right hip that typically mimicked the disease progression of disappearing bone disease. The right femoral head vanished progressively within three months without significant history of infection or trauma. Subsequent bone biopsy of the right femoral head and left tibia condyle confirmed the diagnosis. Total knee replacement was carried out for her left knee. She remained pain free on her left knee. A year later, after confirming by sequential radiographs that the osteolysis had stopped, total right hip replacement was performed. Five years later, she remained pain free and both the arthroplasties were stable.

## Introduction

Disappearing bone disease or Gorham disease is a rare condition, characterised by extensive loss of bony matrix, which is replaced by proliferating thin-walled vascular channel and fibrous tissue^1^. Although it could be monostatic or polyostotic, multicentric involvement is very uncommon^2^. The etiology of this disease is still speculative, the prognosis is unpredictable and the effective treatment is as yet undetermined.

We report our experience in the management of a middle aged patient who presented with severe bone osteolysis of the left tibial medial condyle and right femoral head.

## Case Report

A 46 years old housewife presented with left knee pain of ten months’ duration without significant trauma. She had been treated elsewhere as septic arthritis of the left knee based on the radiological features of severe bone defect of the medical condyle of the left tibia. There was neither history nor clinical appearance suggestive of septic arthritis of the left knee. Results of investigations did not support the diagnosis of infection of her left knee. There was genu varus deformity of her left knee, and she walked with an antalgic gait, occasionally requiring a walking frame for long-distance walking. She had never been hospitalised before. She was given two trials of oral antibiotics empirically but to no avail. Due to the persistent pain that had not resolved with nonsteroidal anti-inflammatory therapy and antibiotics, she was referred to our orthopaedic department.

**Fig. 1:**
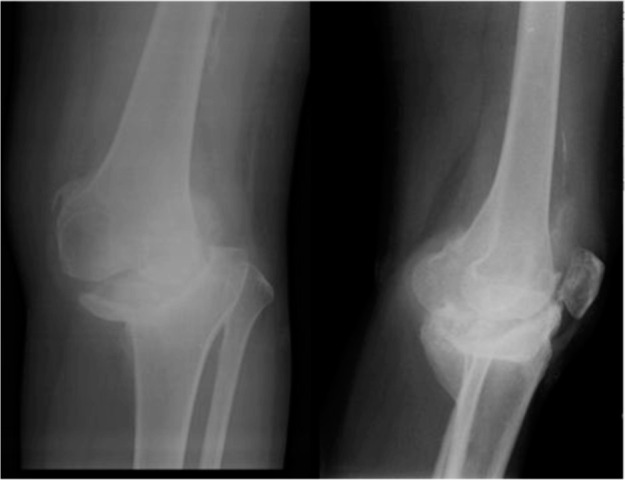
There was a severe defect of medial condyle of the tibia with subluxation of the knee joint. There was sclerosis at the tibia, possibly indicating the boundary of the normal bony architecture with normal osteoblastic activities.

At our centre, we found there was muscular weakness in the lower extremities. Movement of the left knee was painful with limited range of movement of 0° to 90°. The skin in the affected knee was normal without signs of infection, vascular abnormalities or oedema. Peripheral pulses in the left lower limb were normal. The varus deformity of left knee was passively correctable. Radiograph of her left knee showed no progressive changes of the bone defect compared to the bone defect ten months previously. (Fig. 1) Laboratory studies including haematological tests revealed no evidence of metabolic, neoplastic, immunological or infectious aetiology. She was then scheduled for total knee replacement for her left knee, six months later.

**Fig. 2:**
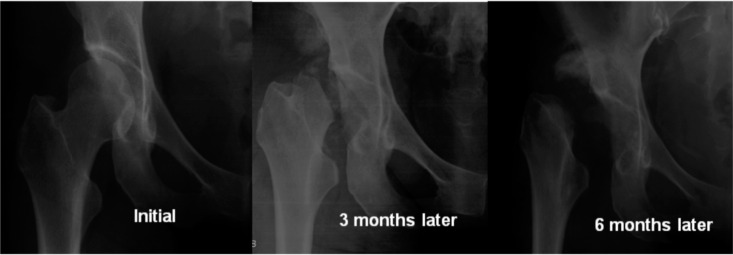
The three radiographs showed that the progression of osteolysis of the femoral head and neck within six months.

However, while awaiting elective surgery, three months after presenting to our centre, she returned with complaint of right hip pain of three weeks duration. The right lower limb was shorter by two centimetres compared to the left lower limb. There was no history of trauma or infection. Radiograph of the right hip revealed complete osteolysis of the femoral head, though a radiograph three months earlier had shown a normal hip joint. (Fig. 2) Laboratory studies again failed to disclose any significant findings for this occurrence as all the haematological tests were within normal limits. Sputum cultures for tuberculosis and Mantoux test were negative. Dual-energy x ray absorptiometry of the spine showed osteopenia. Her family history was non-contributory and no family members had an illness resembling hers.

A CT-guided biopsy of the femoral head was performed. The histopathological results showed few bone trabeculae with many fragments of fibrovascular tissue. A few fragments showed entrapped skeletal muscle and trabeculae of osteoid with osteoblastic rimming. In addition, necrotic cartilage and isolated irregular clusters of amorphous hyaline material were present which stained equivocally with Congo red. There was no cellular evidence of atypia or malignancy. The findings were interpreted as osteocartilaginous necrosis, in keeping with disappearing bone disease.

**Fig. 3:**
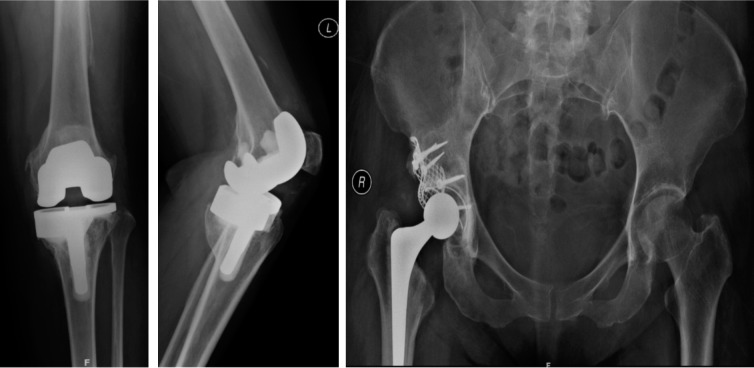
Until date, at 5 years follow-up, the implants are still in situ and there is no progression of bone loss and implant loosening.

We discussed the diagnosis, prognosis and treatment with the patient. After numerous consultations with the patient and her family members, consent was obtained for total knee replacement of her left knee and conservative management of her right hip. We performed arthroplasty for her knee. Histopathological findings from the tissues revealed findings consistent with disappearing bone disease. The patient’s left knee pain was markedly reduced post arthroplasty and she was able to resume her normal activities. There was no radiological evidence of periprosthetic loosening of the tibia implant three months post operation. Due to the good outcome of her left knee, the patient wished to have right hip arthroplasty. A year later, after confirming that the osteolysis had stopped through serial hip radiographs, total hip replacement was performed. At five years post-arthroplasties, she remains pain free and the fixations are stable without any evidence of subsidence. (Fig. 3)

## Discussion

The diagnosis of disappearing bone disease is based on the clinical examination, radiological studies as well as histopathologic examination of the affected area. Dull aching, weakness in the affected limb, swelling and skeletal deformities are predominant symptoms^3^. As in this case, she presented with bony deformity of the left knee which was caused by severe osteolysis of the left medial tibial condyle. The persistent dull aching pain of her right hip prompted us to investigate and subsequently noticed the total resorption of the right femoral head radiologically. However, there were no pathognomonic signs and symptoms that could lead us to the diagnosis of disappearing bone disease. Laboratory studies were of no value in the diagnostic procedure as most of the results in this patient were within normal limits.

The partial or total absence of contiguous bone, tapering of the bony remnants and absence of the sclerosing or osteoblastic reactions are diagnostic radiological features of disappearing bone disease. In this patient, the total resorption of femoral head was evident radiographically. Histopathological examination played an important role to confirm the diagnosis. Heffez *et al* had described histopathological and clinical criteria for diagnosis of massive osteolysis^4^. The histopathological examination of the bone was consistent with massive osteolysis of the bone.

To date, no treatment options have been proven to be beneficial. Numerous methods, including pharmaceutical, surgical, radiation therapy or combinations, have been used to treat this disease^5^. However, the outcome is still controversial. Due to the severe bone defect of the medial condyle of left tibia, we performed total knee arthroplasty with cement augmentation. We did not use radiation therapy in this patient as the results associated with radiation therapy had been equivocal and was only reported as a case report. Besides, due to the advance bony osteolysis of the tibial plateau, the reported therapeutic benefit of radiotherapy in the early stages of disappearing bone disease was hard to justify. Nevertheless, the short term outcome in this patient revealed no progression of the disease or periprosthetic loosening of the implant.

It is very rare to have two different bones disappearing in the same patient. Besides, our strategy of waiting for bone to stop disappearing is in contrast to one of the published case report in which the bone disappeared after the THR causing loosening. In conclusion, disappearing bone disease although regarded as diagnosis of exclusion, poses a great challenge to orthopedist in terms of management, and further research is needed for better understanding of the disease.
